# Identifying cancer mutation targets across thousands of samples: MuteProc, a high throughput mutation analysis pipeline

**DOI:** 10.1186/1471-2105-14-167

**Published:** 2013-05-28

**Authors:** Alireza Hadj Khodabakhshi, Anthony P Fejes, Inanc Birol, Steven JM Jones

**Affiliations:** 1 Genome Sciences Centre, BC Cancer Agency, Suite 100 - 570 West 7th Ave, Vancouver, British Columbia, V5Z 4S6, Canada

## Abstract

**Background:**

In the past decade, bioinformatics tools have matured enough to reliably perform sophisticated primary data analysis on Next Generation Sequencing (NGS) data, such as mapping, assemblies and variant calling, however, there is still a dire need for improvements in the higher level analysis such as NGS data organization, analysis of mutation patterns and Genome Wide Association Studies (GWAS).

**Results:**

We present a high throughput pipeline for identifying cancer mutation targets, capable of processing billions of variations across thousands of samples. This pipeline is coupled with our Human Variation Database to provide more complex down stream analysis on the variations hosted in the database. Most notably, these analysis include finding significantly mutated regions across multiple genomes and regions with mutational preferences within certain types of cancers. The results of the analysis is presented in HTML summary reports that incorporate gene annotations from various resources for the reported regions.

**Conclusion:**

MuteProc is available for download through the Vancouver Short Read Analysis Package on Sourceforge: http://vancouvershortr.sourceforge.net. Instructions for use and a tutorial are provided on the accompanying wiki pages at https://sourceforge.net/apps/mediawiki/vancouvershortr/index.php?title=Pipeline_introduction.

## Background

As Next (Second) Generation Sequencing (NGS) technologies advance, researchers are continually overwhelmed by the massive volume of genomic and transcriptomic sequence data generated. This has significantly shifted the focus of the research from the physical aspects of generating the data toward the analytical aspects of the generated data. Furthermore, while the field is generally at an acceptable point with regards to the primary analysis of the NGS data, such as mapping, assemblies and variant calling, the higher level analysis such as NGS data organization, analysis of mutation patterns and Genome Wide Association Studies (GWAS) are at early stages of development.

As an effort to fill this gap, we proposed an open source variation database [1] template that provides a novel method for collating and searching across the reported results from thousands of NGS samples, as well as rapidly accessing vital information on the origin of the samples. This database package was primarily accompanied with a set of Java application programming interface (API) to perform common functions, such as generation of standard experimental reports and graphical summaries of modifications to genes. In this note we extend our database package with a high throughput mutation analysis pipeline, called MuteProc, to provide more complex analysis on the vast number of variations in the database using a reasonable amount of resources. The most important question that the analysis using this pipeline aims to answer is: “Are there genomic regions that are significantly mutated across certain cancer types?"”

A fundamental design decision underlying the construction of our human variation database template was to make it independent from any annotation set. This allows for efficient scalability and better performance as well as annotation free data storage. However, the mutation analysis pipeline is heavily dependent on genome annotations. This led us to design and implement a stand alone annotation database that ensures the annotation independence requirement of the variation database while providing an efficient way to organize and process various genome annotation resources used by the pipeline. Similar to the human variation database, the annotation database is implemented in PostgreSQL, accompanied by Java API’s to provide necessary interactions with the database.

The human variation database currently stores Single Nucleotide Variations (SNV) and short insertions/deletions (indels) of various NGS protocols. The samples are marked as cancer or normal and the database keeps track of the matching cancer/normal pairs for annotating somatic status of the variations.

## Comparison to existing tools

While many tools are currently available for primary analysis of the sequencing data, there is a shortage of solutions for tertiary analysis, that is the process of extracting insights from the data produced by the upstream analysis steps. Although, one can argue that the wide range of high level analysis does not allow the development of a general purpose tertiary analysis tool, a major tertiary analysis component, that is the identification of common group of variations that affect certain phenotypes in a given population, has yet to be addressed properly.

There are integrated tools designed to provide a cohesive platform for the analysis of next generation sequencing data. These packages include various tools for primary, secondary and tertiary analysis. Here we compare our tool against some of the most widely used tools, that is the Genome Analysis Toolkit (GATK) [2] and the Genome MuSiC [3] with the main focus being on their tertiary analysis functions. The most prominent advantage of MuteProc over these tools is its efficient integration of variation and annotation databases that makes the management of multiple large scale projects as convenient and efficient as possible. This is extremely challenging to achieve using the existing tools since they rely on processing large data files. The GATK package consists of various groups of analytical utilities that mostly deals with primary analysis and Quality Control (QC) steps. In particular, we only one found utility within the GATK that processes the cancer specific variations, i.e. SomaticIndelDetector, and yet this utility can only predict somatic indels in one target sample at a time. Other variation analysis utilities, such as VariantAnnotator, Variant Discovery and Evaluation and Manipulation, either provide primary analysis over individual variants or are limited to analysis over a single sample rather than a cohort of samples which is the prominent feature of MuteProc.

The MuSiC package on the other hand enables collective analysis of mutations across a group of samples, so in this sense MuSiC is a more appropriate benchmark to compare against MuteProc. The MuSiC package consists of a collection of downstream analysis tools designed to (1) apply statistical methods to identify significantly mutated genes, (2) highlight significantly altered pathways, (3) investigate the proximity of amino acid mutations in the same gene, (4) search for gene-based or site-based correlations to mutations and relationships between mutations themselves, (5) correlate mutations to clinical features, and (6) cross-reference findings with relevant databases such as Pfam, COSMIC, and OMIM. Aside from the pathway analysis and the clinical correlation utility, which we aim to include in the later versions, the MuteProc provides all the analytical power of MuSiC with three major advantages: 

1. While the input variations to the MuSiC package are validated or predicted somatic mutations, the MuteProc predicts the somatic mutations from raw mutations generated by variant callers. This is by itself a very challenging task as the mutation set detected by the current variant callers has significant amount of noise. MuteProc predicts somatic variations by filtering tumor mutations against the mutations in matched normal samples, other normal samples in the database and the datasets of known polymorphisms such as DBSNP. The remaining mutations following this stringent filtering stage are then validated by high throughput analysis of the mapped reads in tumor and matching normal samples. Additionally, the mutation frequencies in cancer and normal samples are calculated and the mutations are determined to be synonymous, non-synonymous or non-coding.

2. MuteProc allows mutation analysis over a wide range of annotated genomic regions such as microRNA targets, promoters, enhancers, transcription factor binding sites, regulatory loci and more. In fact any given annotation set can be easily incorporated into the analysis by importing them into the annotation database.

3. MuteProc provides an efficient QC utility for the identified somatic mutations. The QC is carried out by processing the mapped reads at each somatic variation location in tumor and matched normal BAM files and determines whether the variation is likely to be somatic, germline or the result of an artifact. Note that the germline mutations are not excluded from the analysis, instead they are reported separately as in many studies causative predisposing mutations might be of interest. The results of the QC are generated in HTML files that contain the alignment profile of the variations in tumor and matched normal samples placed side by side for easier comparison. These results are incorporated in the final HTML report with provided hyperlink for easy access.

We believe that our mutation analysis package provides some advantages over the existing tools in managing large scale projects involving thousands of samples across multiple cohorts.

## Methods

**Figure 1:**
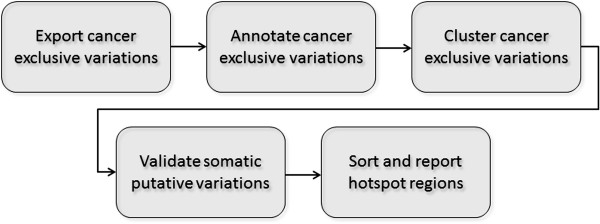
**Validation report.** The major phases of the MuteProc mutation analysis pipeline.

While the utilities in the MuteProc pipeline can be used individually for purposes such as identifying somatic mutation, calculating mutation frequency across a cohort of samples or annotating variations, the main purpose of the pipeline as a whole is to identify genomic regions that are highly mutated across a group of samples (e.g. samples of certain cancer types). These hotspot regions are sorted based on their significant values computed using the multiple test adjusted Fisher’s method that takes into account the mutation rate at each region and the background mutation rate. The mutation analysis is accomplished through the following five phases (See Figure 1.): 

**Phase one (extraction of cancer exclusive variations):** In the first phase, the cancer exclusive variations (i.e. the variations that only occur in the cancer samples in the database) in the target cohort samples are exported from the variation database. The API provides and option to exclude variations that exist in the DBSNP [4] variation set (or any variation set) if they are already imported in the variation database. The efficient organization of the large volume of variations in the HVDB allows this to be performed reasonably quickly. In particular, the cancer SNVs of over two thousand samples, which includes more than 2.5 billion SNV, can be extracted in 22 hours on an eight core Xeon(R) 3.00GHz database server with 64 Gigabytes of main memory. While this is the most time consuming step of the pipeline, the majority of time spent in this phase is utilized for labeling the variations as being synonymous, non-synonymous or non-coding. The output of this phase is a collapsed list of annotated (i.e. silent vs non-synonymous) somatic putative variations. Each variation lists the identifier of the samples that include the variation, thus even at this phase it is easy to find highly recurrent mutations. Indels and SNVs are reported in separate files at this stage, to be merged in the next phase.

**Phase two (annotating the cancer exclusive variations):** In this phase, each cancer exclusive variation is annotated to determine in what region it is located. The set of target annotations are organized in a stand alone annotation database, to ensure fast and effective annotation process. Similar to the HVDB, the annotation database is a PostgreSQL based platform with a set of Java APIs that provides interaction with the database. In addition to ensuring a fast and efficient way of annotating the variations, this database also provides a convenient way to include/exclude the annotation sets of choice. Our annotation database currently stores close to 3 million annotation entries, which include genes, introns, exons, UTRs, transcription factor binding sites, regulatory regions, promoters, enhancers, microRNA targets and Cosmic variations. The software package provides the schema for the database, as well as various APIs for populating the database and retrieving the annotations in a given region. In our case study the annotation process of over 28 million cancer exclusive variations took less than 20 minutes on the same database server platform as the variation database.

**Phase three (clustering the variations):** Clustering of variations is performed in two levels: by *proximity* and by *function*. In the first level the variations are cluster based on their proximity in the genome. This means that the variations that are closely located in the genome (defined by a user specified threshold) are grouped together. Thus, the regions (annotated or novel) that harbor a high rate of variations are identified in this level. In the second level, variation clusters (from level one) that are within the same annotated region are group together. Therefore, mutational hotspots that are functionally related will be identified through the second level clustering. This two-level clustering provides a more informative notion of variation patterns. For instance, one can easily locate regions within a gene that have a high concentration of somatic variations. At the same time novel (unannotated) regions that are significantly mutated can be identified, providing a basis for investigating regions with novel functionality.

In addition to identifying mutational hotspos, regions that are significantly mutated in some types of cancer but not others will be identified in this phase as well. In particular, for each cluster, the number of mutated samples in each group (eg. cancer types) is calculated. Thus, regions that have significantly higher mutation rate in one type of cancer compared to other types can be easily identified. Note that this type of analysis is only viable through processing multiple sample cohorts at the same time and to the best of our knowledge none of the existing GWAS tools provide that.

**Figure 2:**
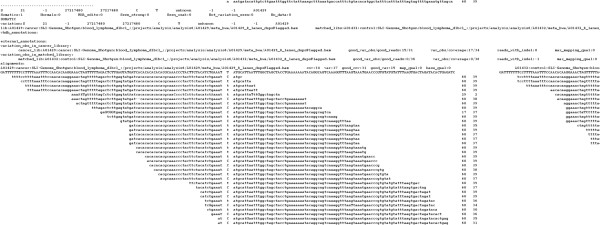
**Validation report.** A snapshot of the report generated by the validation step for the variations in a hotspot region. The alignment profiles of the reads mapped at each variation location is presented in the report. The alignment profile of the matched samples are placed on the side of the tumor samples for a convenient comparison. The two columns beside the aligned reads are the mapping and base calling quality scores, respectively. These reports are accessible through the links provided in the final HTML report for each hotspot region.

**Phase four (validating the somatic variations):** In many cases a variant caller may fail to detect a germline variation in the matched normal sample mainly due to the lack of enough high quality reads covering the variation. Thus, a germline mutation can be incorrectly observed as a somatic mutation. This happens in particular in cases where variations are called on tumor and matched normal samples independently where the reads mapped to the variation allele do not pass the quality thresholds in the normal sample or simply there is no coverage. These incorrectly identified somatic mutations can be detected by processing the mapped reads at the variation location jointly in tumor and normal samples. The validation phase of the MuteProc provides a utility to identify germline or artifact mutations and treat them accordingly (artifacts are removed and germline mutations are processed separately from the somatic mutations). The artifact mutations are detected from the mapping quality of the reads covering the mutation and the quality of the bases of the variation. The minimum quality thresholds are set manually when executing the validation process. The results of this step are reported in HTML files that contain the alignment profile of the variations in tumor and matched normal samples placed side by side for easier comparison (See Figure 2). These results are linked in the final HTML report for easy access. This phase can also be executed in a parallel mode on a computation cluster where a job is dispatched for validating mutations in each region.

**Figure 3:**
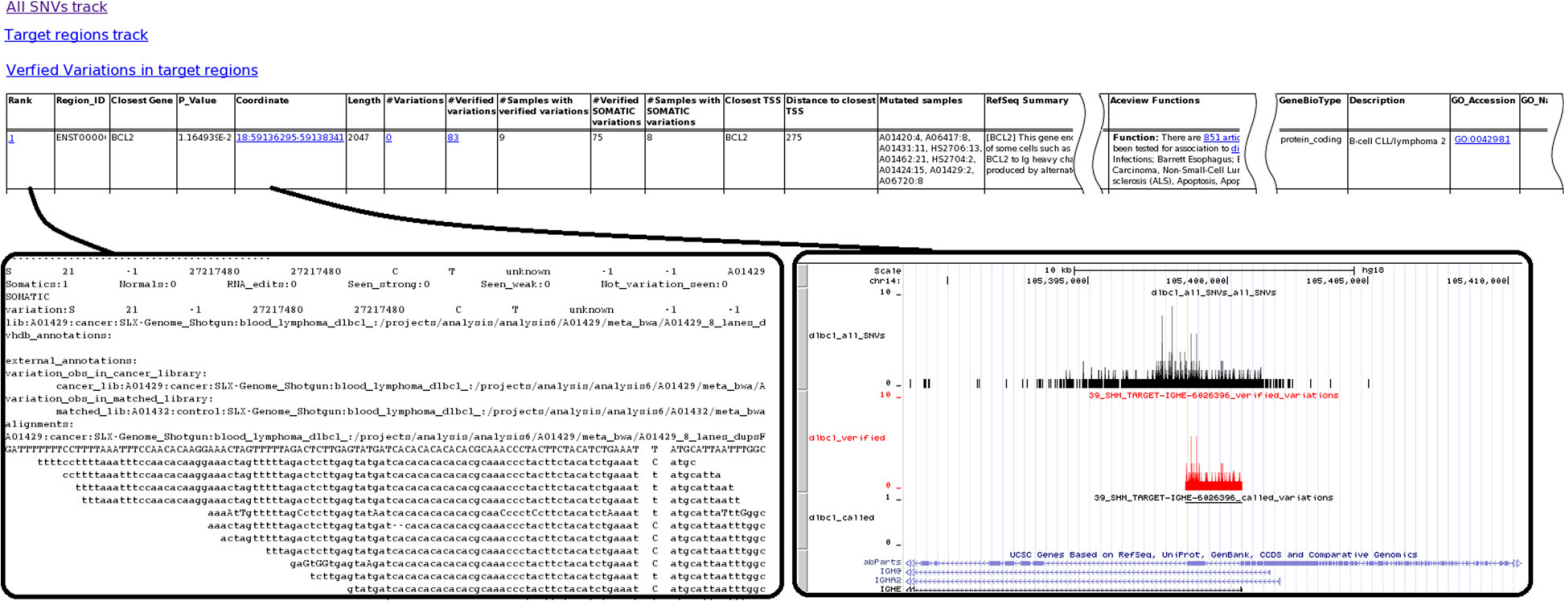
**Final report.** A snap shot of the final report generated by the pipeline. The links in the “rank” column point to the variation QC report for the corresponding region. The three links on the top of the report, that is “All SNVs track”, “Target regions track” and “Verified Variations in target regions”, uploads the variation locations as custom tracks in the UCSC genome browser. Once these tracks are uploaded, clicking on the links in the “Coordinate” column browse to the associated region in the UCSC genome browser where the variations are visible in the loaded variation tracks.

**Phase five (sorting and reporting):** Once the clusters of variations have been identified, they are assigned a p-value that reflects the statistical significance of the mutations in the region. Briefly, the p-values are calculated by incorporating the rate of the observed mutation in the region and the expected mutation rate on a random basis using a Binomial statistical test function. The resulting p-values are then corrected for multiple testing using the Benjamini Multiple Testing Correction approach. The clusters are sorted by the computed significance values, and the results are presented in a hyper-linked HTML formatted table (See Figure 3). Each hotspot region in the final report is augmented by a summary of functional annotations from various resources, including AceView [5], Biomart [6] and Ensembl [7]. The HTML report also includes hyper-links to the variation location in the form of UCSC browser custom tracks that can be easily viewed in the UCSC browser. This provides a convenient way to zoom in on the mutated regions and, view further annotations for the affected regions.

The details of each API including the input parameters, input and output formats and, how to run them is fully explained in the wiki pages at https://sourceforge.net/apps/mediawiki/vancouvershortr/index.php?title=Pipeline_introduction. We have also provided a self-contained tutorial that explains all the steps from setting up and populating the variation and the annotation databases to running the mutation analysis pipeline. This tutorial comes with a small set of RNA-seq variations for 10 prostate samples and their matched normal samples along with shell scripts that perform each step of the pipeline for the sample dataset.

## Results

As a proof of concept, using MuteProc, we performed a Genome Wide Analysis on 40 whole genome Diffuse Large B-Cell Lymphoma tumour/normal sample pairs aiming to identify regulatory regions that are significantly mutated in our cohort. While detecting non-synonymous driver mutations has been the focus of the majority of cancer genome studies, recent studies show that non-coding mutations may drive cancer as well [8]. The reason that non-coding mutations have been left out from most of the cancer genome analysis is the challenge of discovering their role in cancer. However, if a large cohort of whole genome samples is available to derive statistically significant conclusions, it is possible to identify candidate non-coding regions that may influence cancer development.

The results of MuteProc analysis on our DLBCL cohort showed that the promoters and UTRs of several genes are mutated at rates dramatically higher ( ∼1000-fold) than other mutated regions. Further analysis of the mutations in these regions revealed that they have similar characteristics to those of aberrant aberrant somatic hypermutations (aSHM) in DLBCL. Somatic hypermutation (SHM) in the variable region of immunoglobulin genes (IGV) naturally occurs in a narrow window of B cell development to provide high-affinity antibodies. However, SHM can also aberrantly target proto-oncogenes and, cause genome instability [9]. Several studies in the past decade collectively reported twelve genes that are targets of aSHM in Lymphoma [9-11], however it was speculated that far more genes undergo aSHM in Lymphoma [9]. Intriguingly, all of these known aSHM targets appeared at the of the top of our list, indicating the accuracy of our analysis. Through the results of this analysis we were able to discover over 30 novel targets of aSHM in DLBCL. For further details of this study please refer to our manuscript in Oncotarget [12].

## Discussion

In this section we discuss the limiting aspects of MuteProc and put forward our plan to make improvements in later versions.

While SNVs and Indels comprise most of the genomic variations, structural variations, copy number variation as well as more complex rearrangements have significant source of biological and clinical relevance in diseases in particular cancer. Thus, we are in the process of including these variations in our high throughput analysis pipeline. The design and implementation of a consortium of databases of genomic data, including copy number variation, LOH events, structural variations and expression data has been completed at the BC Genome Sciences Centre and we are now in the process of populating these databases [13]. The next step will be to integrate these databases in the MuteProc pipeline. While this is a challenging task especially with respect to performance given the vast amount of data to be processed, the results will be rewarding. It will enable us to discover meaningful correlations between statistically significant mutations of various types and the biological phenotypes.

Identifying altered pathways and correlating clinical outcomes with variations are other components that are currently missing in the pipeline. Although, the current database schema stores the clinical outcome data and the software package has a stand alone utility for processing this information, this analysis is yet to be incorporated in the pipeline.

## Conclusions

We described MuteProc, a high throughput pipeline for collective analysis of mutations in cohorts of NGS samples. A key advantage of MuteProc is its integration with a variation database that makes the management of multiple projects involving thousands of NGS samples as convenient as possible. The variation database that MuteProc is tied to at the BC Genome Sciences Centre currently holds over 2.5 billion SNVs and Indels in over 4000 NGS samples. MuteProc is able to efficiently process this vast volume of variations and identify mutational hotspots across hundreds of samples. We believe that the research community will benefit from this open source package in analyzing the ever increasing NGS data.

## Competing interests

The authors declare that they have no competing interests.

## Authors’ contributions

AHK performed the research, developed analysis software and wrote the paper. APF contributed to the software development. IB and SJM. Jones contributed to conceiving and designing the research. All authors read and approved the final manuscript.
